# Isolated Striatocapsular Infarcts after Endovascular Treatment of Acute Proximal Middle Cerebral Artery Occlusions: Prevalence, Enabling Factors, and Clinical Outcome

**DOI:** 10.3389/fneur.2017.00272

**Published:** 2017-06-19

**Authors:** Johannes Kaesmacher, Thomas Huber, Manuel Lehm, Claus Zimmer, Kathleen Bernkopf, Silke Wunderlich, Tobias Boeckh-Behrens, Nathan W. Manning, Justus F. Kleine

**Affiliations:** ^1^Department of Diagnostic and Interventional Neuroradiology, Klinikum rechts der Isar, Technische Universität München, Munich, Germany; ^2^Institute for Clinical Radiology, Ludwig-Maximilians-University Hospital Munich, Munich, Germany; ^3^Department of Neurology, Klinikum rechts der Isar, Technische Universität München, Munich, Germany; ^4^Florey Institute of Neuroscience and Mental Health, University of Melbourne, Parkville, VIC, Australia; ^5^Department of Neuroradiology, Charité, Berlin, Germany

**Keywords:** stroke, striatocapsular infarcts, thrombectomy, endovascular, basal ganglia, prevalence

## Abstract

**Background:**

Striatocapsular infarcts (SCIs) are defined as large subcortical infarcts involving the territory of more than one lenticulostriate artery. SCI without concomitant ischemia in the more distal middle cerebral artery (MCA) territory [isolated SCI (iSCI)] has been described as a rare infarct pattern. The purpose of this study was to assess the prevalence of iSCI in patients treated with endovascular thrombectomy (ET), to evaluate baseline and procedural parameters associated with this condition, and to describe the clinical course of iSCI patients.

**Methods:**

A retrospective analysis of 206 consecutive patients with an isolated MCA occlusion involving the lenticulostriate arteries and treated with ET was performed. Baseline patient and procedural characteristics and ischemic involvement of the striatocapsular and distal MCA territory [iSCI, as opposed to non-isolated SCI (niSCI)] were analyzed using multivariate logistic regression models. Prevalence of iSCI was assessed, and clinical course was determined with the rates of substantial neurological improvement and good functional short- and mid-term outcome (discharge/day 90 Modified Rankin Scale ≤2).

**Results:**

iSCI was detected in 53 patients (25.7%), and niSCI was detected in 153 patients (74.3%). Successful reperfusion [thrombolysis in cerebral infarction (TICI) 2b/3] [adjusted odds ration (aOR) 8.730, 95% confidence interval (95% CI) 1.069–71.308] and good collaterals (aOR 2.100, 95% CI 1.119–3.944) were associated with iSCI. In successfully reperfused patients, TICI 3 was found to be an additional factor associated with iSCI (aOR 5.282, 1.759–15.859). Patients with iSCI had higher rates of substantial neurological improvement (71.7 vs. 37.9%, *p* < 0.001) and higher rates of good functional short- and mid-term outcome (58.3 vs. 23.7%, *p* < 0.001 and 71.4 vs. 41.7%, *p* < 0.001). However, while iSCI patients, in general, had a more favorable outcome, considerable heterogeneity in outcome was observed.

**Conclusion:**

High rates of successful reperfusion (TICI 2b/3) and in particular, complete reperfusion (TICI 3) are associated with iSCIs. The high prevalence of iSCI in successfully reperfused patients with good collaterals corroborates previous concepts of iSCI pathogenesis. iSCI, once considered a rare pattern of cerebral ischemia, is likely to become more prevalent with increases in endovascular stroke therapy. This may have implications for patient rehabilitation and pathophysiological analyses of ischemic damage confined to subcortical regions of the MCA territory.

## Introduction

Striatocapsular infarcts (SCIs) are defined as large subcortical infarcts in the territory of the lenticulostriate perforator arteries (1). They are caused by a simultaneous blockage of multiple neighboring perforators owning to a transient or permanent blockade of the proximal middle cerebral artery (MCA) or the carotid T (1–5). Thus, their pathogenesis and underlying risk factors differ from those of lacunar infarcts that are caused by an acute disruption of a single perforator due to hypertensive lipohyalinosis (6–8).

Because the striatocapsular territory lacks a collateral supply, the development of ischemia in the striatocapsular regions following proximal MCA occlusions is determined by the exact thrombus location and subsequent involvement of the perforators (9, 10). In contrast, the variability in preservation of the peripheral hemispheric tissue following large vessel occlusion (LVO) is mainly attributable to different degrees of collateralization (11, 12) and highly dependent on the level of reperfusion (8, 13–16). This suggests that while the volume of more peripheral, cortical infarct depends on both, the extent of reperfusion and the time until reperfusion, striatocapsular infarction is likely to be time and reperfusion independent (10). Isolated striatocapsular infarction (iSCI) should then be encountered more commonly in the setting of highly effective reperfusion therapy.

Prior to the advent of effective reperfusion therapy iSCI was rarely observed, with a reported prevalence in large published stroke registries ranging from less than 0.01 to 6% (1, 4, 17–21). Successful reperfusion is considered necessary for the development of iSCIs (22), and a recent study suggesting iSCI is more prevalent in patients treated with intravenous tPA (IV-tPA) supports this notion (23). However, IV-tPA has limited efficacy in LVOs (24, 25). Endovascular thrombectomy (ET) is now established as a highly effective reperfusion strategy for LVOs of the anterior cerebral circulation (26). To date, iSCIs have not been assessed previously together with angiographically confirmed reperfusion or more broadly in ET. This study aims to investigate the prevalence of iSCI in isolated proximal MCA occlusions treated with ET. Further, we assessed baseline and procedural characteristics associated with its occurrence and described the clinical course of iSCI patients. As ET of LVOs is expected to increase, this may have important implications for not only acute stroke management but also patient rehabilitation and may give insights into the pathophysiological processes underlying isolated ischemia of distinct parts of the caudate nucleus, striatum, and/or the internal capsule.

## Materials and Methods

### Patient Population

**Figure 1:**
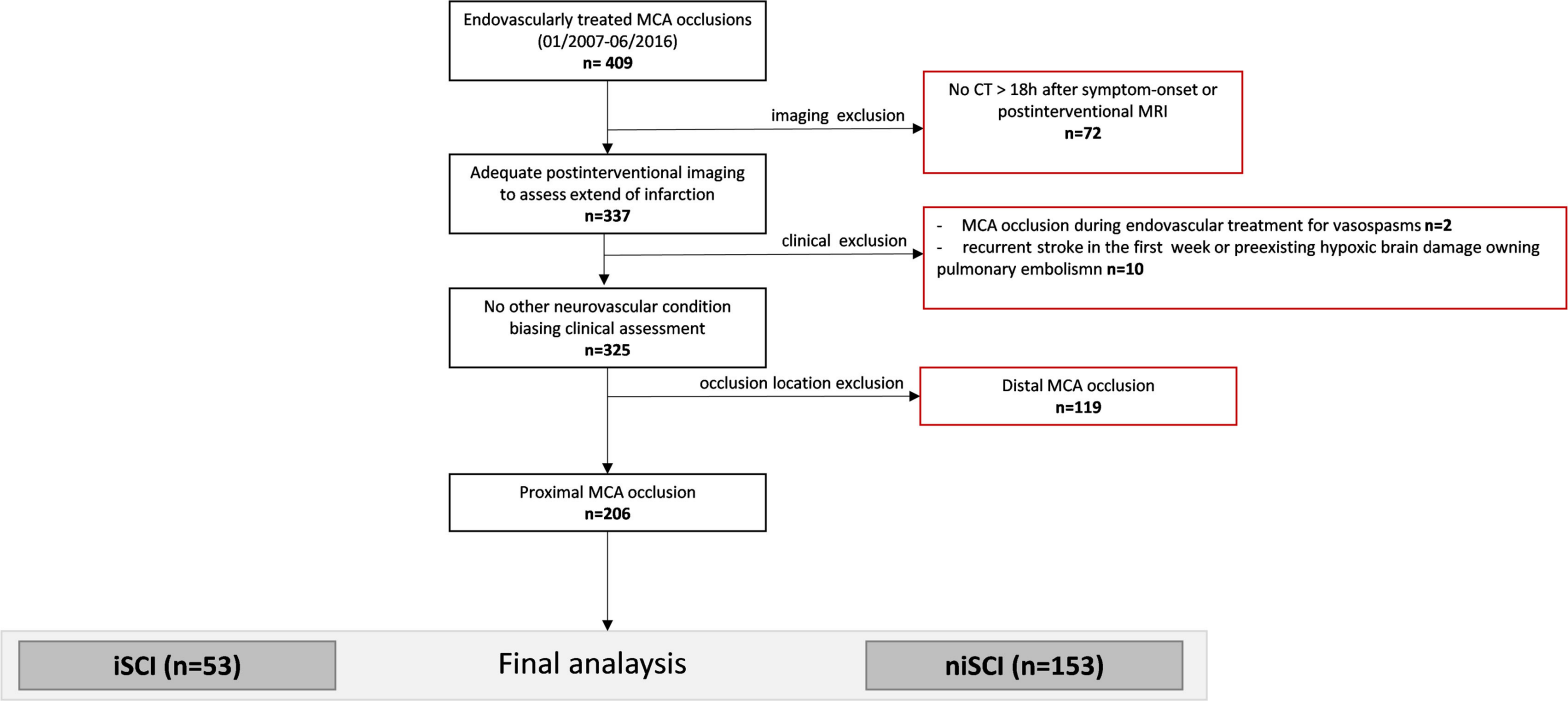
Study flowchart. MCA, middle cerebral artery; iSCI, isolated striatocapsular infarct; niSCI, non-isolated striatocapsular infarct.

Consecutive patients with isolated MCA occlusion who were subjected for ET at our institution between 01/2007 and 06/2016 were included in the screening of potentially eligible patients (*n* = 409, see study flowchart displayed in Figure 1). All patients with adequate imaging to assess the extent of the infarction (defined as either MRI at day 3 or CT >18 h after symptom onset) were included (*n* = 337). Two patients in whom an MCA occlusion occurred during the treatment of vasospasm were excluded, as were nine patients in whom recurrent stroke biased clinical assessment and one patient in whom hypoxic brain damage occurred following pulmonary artery embolism. Further, we excluded patients presenting with a distal MCA occlusion, defined as sparing of the lenticulostriate arteries. The final analysis included 206 patients. The study was performed with the ethics approval from the local ethics committee. Written informed consent was waived due to its retrospective design according to the institutional guidelines.

### Endovascular Therapy

According to our institutional guidelines, all stroke patients with CTA-proven LVO were eligible for ET in the absence of evidence of extensive early infarction on CT (involving more than one-third of the MCA territory) and if the time from symptom onset to treatment was less than 6 h. No other image-guided selection or age limit was applied. Preinterventional intravenous recombinant tissue plasminogen activator (IV rtPA) was administered as bridging therapy in the absence of contraindications (*n* = 137; 66.5%). Procedures were performed under general anesthesia or conscious sedation. In the vast majority of patients (*n* = 167; 76.6%), thrombectomy was performed with the use of stent retrievers. Other applied techniques consisted of sole aspiration (*n* = 8) or a combination of aspiration and stent retriever (*n* = 10). In six patients, a permanent stent was implanted because of an underlying high-grade MCA stenosis. Eighteen patients were treated with earlier generation devices/techniques (e.g., the MERCI retriever or intra-arterial rtPA). Three patients with spontaneous vessel recanalization, as revealed by first diagnostic DSA runs, were also included in the analysis.

### Imaging Assessment

**Figure 2:**
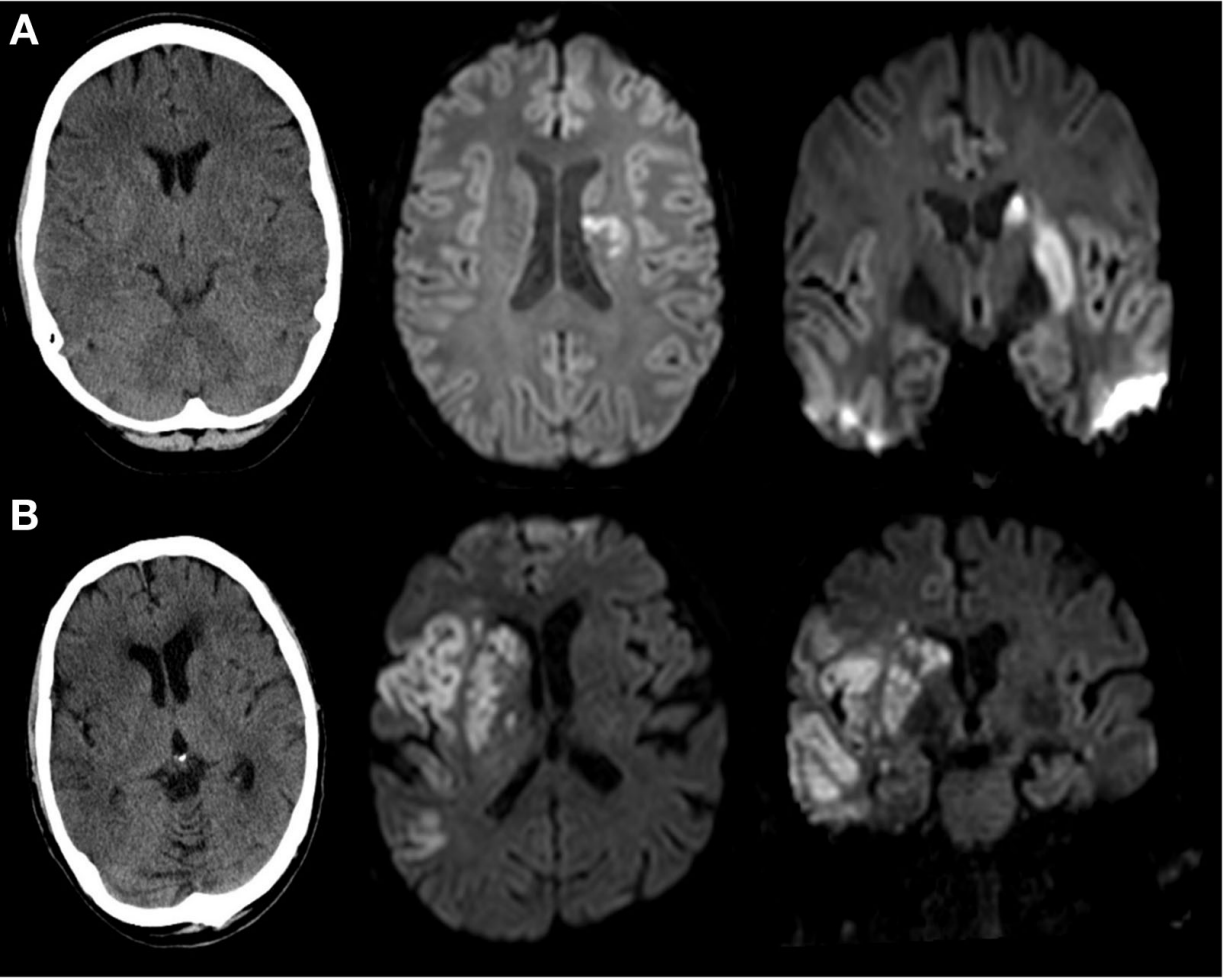
Imaging examples of isolated (iSCI) and non-isolated striatocapsular infarct (niSCI). Left column, non-contrast CT; middle column, axial diffusion-weighted imaging; right column, coronar diffusion-weighted imaging. **(A)** iSCI; **(B)** niSCI.

Two neuroradiologists independently classified striatocapsular territory infarction on postinterventional MRI (*n* = 104, 50.5%) or non-contrast CT (NCT, *n* = 102, 49.5%) blinded to all clinical data. Postinterventional NCT was usually performed at 12 h postintervention and at day 3 after endovascular treatment. Median time from intervention to MRI was 3 days [interquartile range (IQR) 1–4 days]. A consensus read was used in cases of disagreement. With exception of small punctual embolic ischemia, as evaluated on MRI, the absence of acute ischemic lesions other than striatocapsular lesions was defined as isolated SCI (iSCI, Figure 2A). Concomitant involvement of the more peripheral MCA territory was rated as non-isolated SCI (niSCI, see Figure 2B). Symptom-onset-to-treatment time (SOTT) and symptom-onset-to-reperfusion time (SORT) were defined as time interval between symptom onset to first intracranial DSA series or substantial reperfusion, respectively. Thrombolysis in cerebral infarction (TICI)-graded reperfusion success was assessed by two neuroradiologists independently, with TICI 2b defined as reperfusion of more than 66% of the initially involved territory, according to the original TICI scale (27). Successful reperfusion was defined as TICI 2b/3. TICI 3 reperfusion was referred to as complete reperfusion. Thrombus location within the MCA was dichotomized in proximal and distal occlusion (involving/sparing the lenticulostriate arteries, respectively). Only proximal occlusions were included into the analyses (see Patient Population). Hemorrhagic infarctions (HIs) and parenchymal hematomas (PHs) on postinterventional imaging were evaluated according to the ECASS criteria (28). Preinterventional collateral grading was performed using a 4-step grading system (0–3) as applied by the MR CLEAN investigators (29, 30). In short, 0 refers to 0% filling of the occluded territory, 1 refers to poor collaterals with >0% but ≤50% filling of the occluded territory, 2 refers to moderate collaterals with >50% but <100% filling of the occluded territory, while 3 describes good collaterals with 100% filling of the occluded territory. Only mothership patients with available high quality single-phase CTA were graded (*n* = 108, 52.5%).

### Clinical Assessment

Baseline and laboratory findings were extracted by reviewing patients’ medical charts. Clinical outcome was assessed by a qualified neurologist using the National Institutes of Health Stroke Scale (NIHSS), Modified Rankin Scale (mRS), and Barthel index scores at the day of discharge (NIHSS-DIS, mRS-DIS, Barthel-DIS). A maximum NIHSS score was assigned to a fatal outcome during the acute hospital stay. “Substantial neurologic improvement” was prespecified as compound criterion of either (i) difference between admission NIHSS and NIHSS-DIS ≥8 (ΔNIHSS ≥8), or (ii) NIHSS-DIS ≤1, as this dichotomization has been shown to be sensitive to detecting therapy effects in acute ischemic stroke (31). If available, short- (*n* = 187, 90.8%) and mid-term functional outcome (*n* = 150, 72.8%) was assessed at discharge/day 90, and mRS≤ 2 was defined as “good functional outcome.”

### Statistical Analysis

Interrater reliability was evaluated using Cohen’s Κ. Continuous variables and frequency counts were compared using standard statistical measures (Welch’s *t*-test, Mann–Whitney *U*-test, and Fisher’s exact test). Data are shown as median and IQR or mean ± SD, if normally distributed. A second stroke after the index stroke was treated as an independent observation if the time difference between the two ischemic events was sufficient to allow adequate clinical distinction (≥6 months), as detected in three patients that were included in the analysis. Logistic regression modeling for factors associated with iSCI was performed adjusting for age, and variables with *p* < 0.1 in univariate logistic regression. Logistic regression modeling was performed for all patients with complete data of the variables included and for a subset of patients in whom successful reperfusion was reached. Only in the latter model, the term TICI 3 reperfusion was included because of high collinearity with the binary variable “successful reperfusion.” Data of logistic regression models are displayed as adjusted odds ratio (aOR) and 95% confidence interval (95% CI).

## Results

### Study Population

Two hundred and six patients were included in the final analysis (119 women; 57.8%). Mean age was 71.1 ± 14.9 years. Median NIHSS at presentation was 15 (IQR 12–18). Good functional outcomes at discharge and at 90-day follow-up were noted in 32.6% (*n* = 61/187) and 50.0% (*n* = 75/150), respectively. In approximately one-quarter of the patients presenting with an acute proximal MCA occlusion (*n* = 53/206), the peripheral MCA territory was spared leading to an iSCI. Subsequently, niSCI was noted in 153 patients. Interrater reliability was excellent for both classification of iSCI (Κ > 0.9) and evaluation of reperfusion success using the TICI score (Κ > 0.9).

### Factors Associated with iSCI Occurrence: Comparison of iSCI vs. niSCI

**Figure 3:**
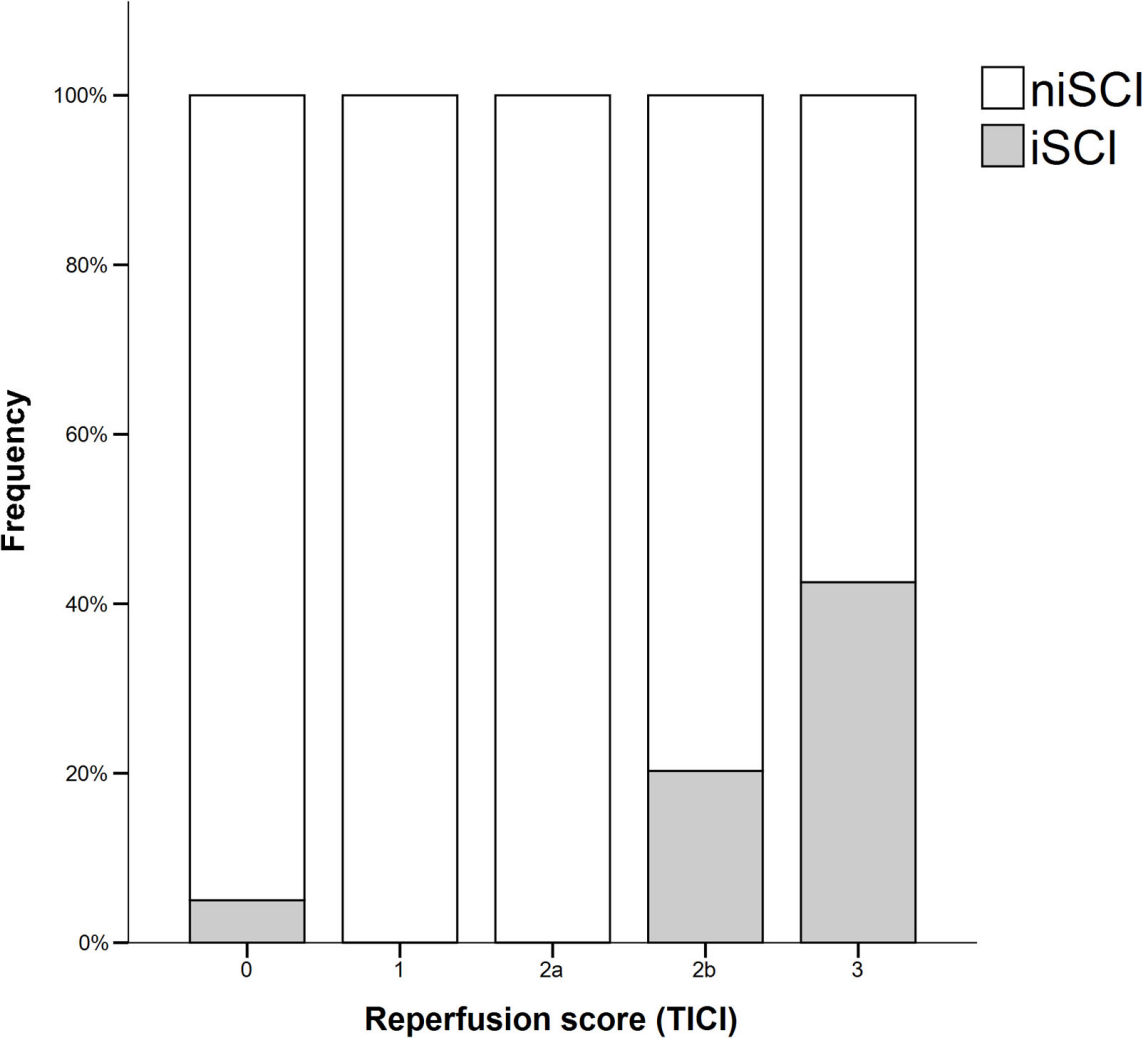
Frequency of isolated striatocapsular infarcts stratified with respect to reperfusion grades. iSCI, isolated striatocapsular infarct; niSCI, non-isolated striatocapsular infarct; TICI, thrombolysis in cerebral infarction.

Prevalence of iSCI varied between the different grades of reperfusion (see Figure 3, *p* < 0.001). Correspondingly, iSCI was observed more often in successfully reperfused patients (TICI 2b/3) as opposed to patients in whom successful reperfusion could not be achieved (32.3 vs. 1.9%, *p* < 0.001). Furthermore, iSCI occurred more often after complete reperfusion (TICI 3) than after TICI 2b reperfusion (42.2 vs. 21.8%, *p* = 0.007). Comparing baseline and procedural characteristics, patients with iSCIs tended to have a lower baseline NIHSS scores (median 14 vs. 15, *p* = 0.063, see Table 1), better collaterals [median grade 2 (IQR 2–3) vs. 2 (IQR 1–2), *p* = 0.045] and had higher rates of successful (TICI 2b/3; 98.1 vs. 71.2%; *p* < 0.001) and complete (TICI 3; 60.4 vs. 39.6%; *p* < 0.001) reperfusions. Furthermore, IV rtPA bridging therapy tended to be more frequently administered in iSCI patients (77.4 vs. 62.7%, *p* = 0.063). Both groups were comparable regarding risk factor profile, age, and admission laboratory findings. Symptom-onset to treatment and symptom-onset to reperfusion did not differ between both groups (median 212 vs. 210 min and median 263 vs. 277 min for iSCI vs. niSCI, respectively). In multivariate logistic regression analysis, successful reperfusion (TICI 2b/3) (aOR 8.730, 95% CI 1.069–71.308) and good collaterals (aOR 2.100, 95% CI 1.119–3.944) were associated with iSCI (see Table 2). In successfully reperfused patients, TICI 3 reperfusions (aOR 5.282, 1.759–15.859) were found as an additional factor associated with iSCI. When additionally entered in the model, symptom-onset to reperfusion was not associated with the occurrence of iSCI (aOR 1.001, 95% CI 0.997–1.005).

**Table 1:** Baseline and procedural characteristics.

	iSCI (*n* = 53)	niSCI (*n* = 153)	*p*
Age	72.3 ± 16.7	70.6 ± 14.3	0.532
Sex, female	60.4% (32)	56.9% (87)	0.747
Diabetes	11.3% (6/53)	21.3% (32/150)	0.151
Hypertension	69.8% (37/53)	75.3% (113/150)	0.469
Atrial fibrillation	54.7% (29/53)	50.7% (76/150)	0.635
Previous stroke	13.2% (7/53)	19.3% (29/150)	0.404
Admission glucose (mg/dl)	122 (109–147)	128 (109–156)	0.469
Baseline NIHSS	14 (12–17)	15 (12–18)	0.063
IV rtPA	77.4% (41)	62.7% (96)	0.063
Wakeup stroke	3.8% (2)	7.2% (11)	0.522
Collaterals (0–3) *n* = 108	2 (2–3)	2 (1–2)	0.045*
SOTT (min)	212 (168–263)	210 (165–270)	0.633
SORT (min)	263 (191–311)	277 (226–331)	0.287
Successful reperfusion (TICI 2b/3)	98.1% (52)	71.2% (109)	<0.001**
Complete reperfusion (TICI 3)	66.0% (35)	31.4% (48)	<0.001**
Modality used for classification, MRI	60.4% (32)	47.1% (72)	0.112

*Data are displayed as % (*n*) or median (interquartile range)*.

*iSCI, isolated striatocapsular infarct; niSCI, non-isolated striatocapsular infarct; SOTT, symptom-onset-to-treatment time; SORT, symptom-onset-to-reperfusion time; TICI, thrombolysis in cerebral infarction; IV rtPA, intravenous recombinant tissue plasminogen activator; NIHSS, National Institutes of Health Stroke Scale*.

*p < 0.05.

**p < 0.01.

**Table 2:** Multivariate logistic regression.

	Adjusted odds ratio	95% Confidence interval	*p*
**Patients with available collateral data (***n*** = 108)**			
Age	1.033	1.000–1.068	0.051
Intravenous recombinant tissue plasminogen activator (IV rtPA)	1.203	0.450–3.217	0.713
Baseline National Institutes of Health Stroke Scale (NIHSS)	1.060	0.937–1.200	0.352
Successful reperfusion [thrombolysis in cerebral infarction (TICI) 2b/3]	8.730	1.069–71.308	0.043*
Collaterals	2.100	1.119–3.944	0.021*
**Patients with successful reperfusion and available collateral data (***n*** = 86)**			
Age	1.011	0.881–1.161	0.872
IV rtPA	1.197	0.418–3.428	0.737
Baseline NIHSS	1.011	0.881–1.161	0.872
TICI 3 reperfusion	5.282	1.759–15.859	0.003**
Collaterals	2.385	1.135–5.010	0.022*

*p < 0.05.

**p < 0.01.

*Adjusted odds ratios for collaterals refer to every grade increase*.

### Clinical Outcome

**Figure 4:**
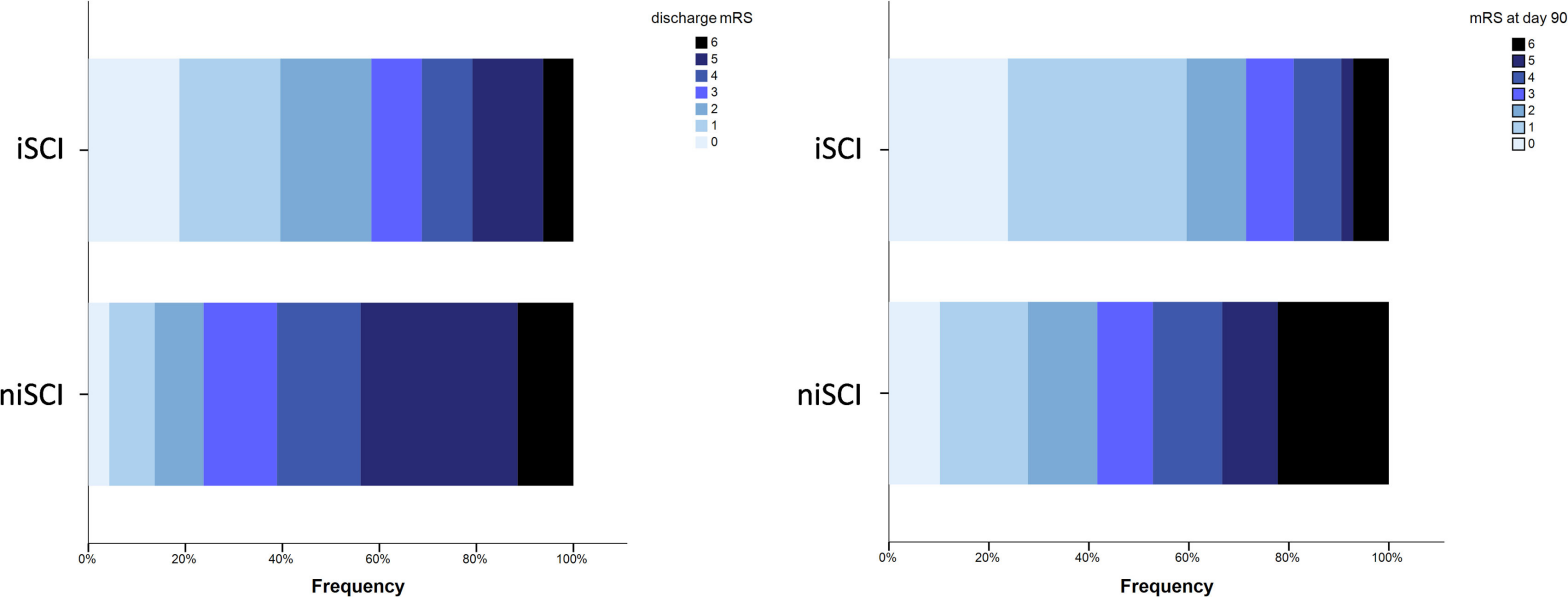
Short- and mid-term functional outcome of patients with iSCIs vs. niSCIs. Rates of good functional outcome were significantly higher in iSCI patients (*p* < 0.001). Discharge/day 90 mRS was available for 48/53 and 42/53 iSCI patients and 139/153 and 108/153 niSCI patients. iSCI, isolated striatocapsular infarct; niSCI, non-isolated striatocapsular infarct; mRS, Modified Rankin Scale.

**Figure 5:**
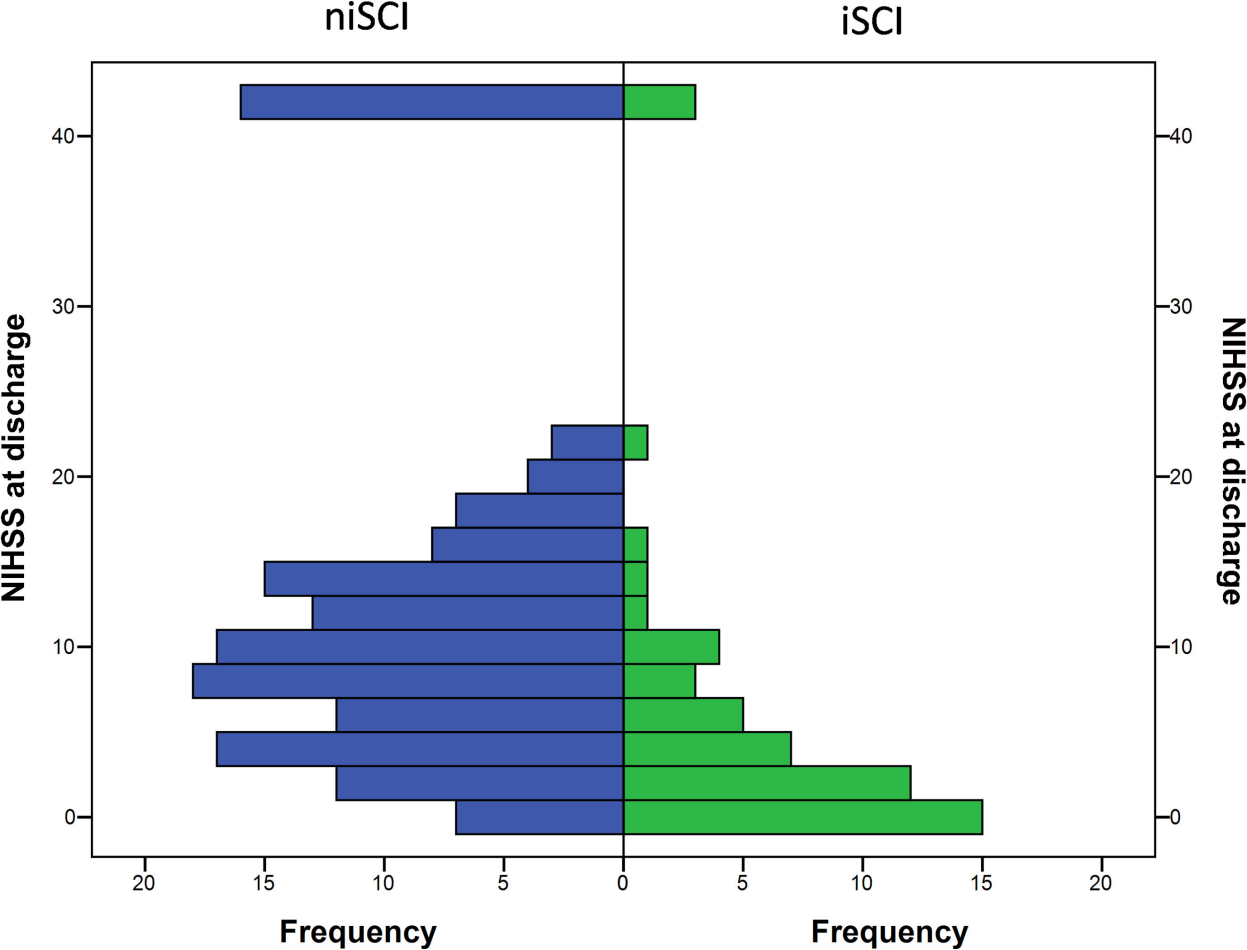
Neurological symptom severity of patients with iSCIs vs. niSCIs at the day of discharge. iSCI, isolated striatocapsular infarct; niSCI, non-isolated striatocapsular infarct.

No significant difference was observed for the incidence of PH (1.9 vs. 5.2%, *p* = 0.452), HI (32.1 vs. 33.3%, *p* = 1.000) or in-hospital mortality (5.7 vs. 10.5%, *p* = 0.413) when comparing iSCI and niSCI patients. Patients with iSCI had higher rates of good clinical outcome, specifically, higher rates of substantial neurologic improvement (71.7 vs. 37.9%, *p* < 0.001, see Table 3), higher median Barthel index scores at discharge (median 45 vs. 15, *p* < 0.001) and higher rates of good functional short- and mid-term outcome (58.3 vs. 23.7%, *p* < 0.001 and 71.4 vs. 41.7%, *p* < 0.001, respectively, see Figure 4). However, while iSCI patients in general had a more favorable outcome, considerable heterogeneity in outcome was observed (see Figures 4 and 5).

**Table 3:** Outcome.

	iSCI (*n* = 53)	niSCI (*n* = 153)	*p*
HI	32.1% (17)	33.3% (51)	1.000
PH	1.9% (1)	5.2% (8)	0.452
In-hospital mortality	5.7% (3)	10.5% (16)	0.413
NIHSS-DIS	2 (0–7)	10 (5–15)	<0.001**
Substantial neurologic improvement	71.7% (38)	37.9% (58)	<0.001**
Barthel-DIS	45 (25–75)	15 (5–45)	<0.001**
mRS-DIS	2 (1–4)	4 (3–5)	<0.001**
Good functional short-term outcome (mRS-DIS ≤2)	58.3% (28/48)	23.7% (33/139)	<0.001**
d90 mRS	1 (1–3)	3 (1–5)	<0.001**
Good function mid-term outcome (d90 mRS ≤2)	71.4% (30/42)	41.7% (45/108)	0.002**

*Data are displayed as % (*n*) or median (interquartile range)*.

*HI, hemorrhagic infarction; PH, parenchymal hematoma; DIS, discharge; d90, day 90 after admission; iSCI, isolated striatocapsular infarct; niSCI, non-isolated striatocapsular infarct; NIHSS, National Institutes of Health Stroke Scale; mRS, Modified Rankin Scale*.

**p < 0.01.

## Discussion

This study demonstrates strong evidence for an association between the extent of reperfusion and the development of iSCI in MCA occlusions. This is the first study of iSCI reported to date in a large cohort of patients treated with ET in a single center. These results support the strong dependency of iSCI on vessel recanalization (23) and good collaterals and thus concur with current concepts of iSCI pathogenesis (3, 4, 32). iSCIs demonstrated strong evidence for an association with good functional outcome. Together, both the association with reperfusion and the association with good functional outcome may, at least in part, explain why reperfusion has recently been described as the critical driver of outcome in ET (26). This suggests that iSCIs may provide a distinct imaging correlate, which reflects the treatment superiority of ET in proximal MCA occlusions (33–37).

Our results further support the notion that achieving quality reperfusion and, at best, complete (TICI 3) reperfusion may be the most crucial determinant of favorable outcome (38, 39). The striatocapsular region may undergo infarction relatively early; owing to a lack of collateral pathways (9, 10). However, by achieving complete (TICI 3) reperfusion, there is greater likelihood of preserving the penumbral cortical tissue in comparison to less complete degrees of reperfusion.

At first, the lack of evidence for an association between iSCI and time to treatment may seem counterintuitive. Reduced time to groin puncture and time to reperfusion have demonstrated very strong evidence for associations with improved functional outcome (40, 41). However, it is important to note that patients may achieve good functional outcomes (mRS 0–2) while suffering more extensive cerebral infarctions than iSCI. Indeed, the magnitude of effect of reduction in time to groin puncture or time to reperfusion in the recent thrombectomy trials is remarkably small (1–4% per 30 min reduction) (41). This may be explained by the incremental effect of preserving cortical penumbra rather than the striatocapsular tissue. Interestingly van Overbeek and colleagues also found no evidence for an association between time to treatment with IV-tPA and iSCI (23). This time insensitivity may be explained by the *sine qua nons* condition of robust collaterals required in the pathogenesis of iSCI, which is supported by the presented data. Patients without sufficient collateral supply may recruit cortical penumbra into the ischemic core early; precluding these patients from iSCI categorization. Patients with sufficient collateral supply to the cortical penumbra may be relatively insensitive to time, at least within the current study’s treatment window (123–717 min) and therefore achieve iSCI in the presence of quality reperfusion. Previous work has demonstrated that penumbral recruitment by the ischemic core occurs at a very slow rate in the presence of good collaterals (11), and the present study supported evidence that the specific association between collaterals and iSCI also holds true in the setting of ET.

To the best of our knowledge four major pathophysiologic processes have been proposed to cause iSCIs: (1) emboli to the proximal MCA (14, 42); (2) triangular-shaped carotid T occlusion allowing cortical supply (1); (3) extracranial carotid artery occlusion with either presumed emboli to the proximal MCA or hemodynamic alterations leading to deep territory infarction (4, 32); (4) atherosclerotic disease of the MCA with concomitant *in situ* thrombosis or other isolated MCA abnormalities (dissection, vasculitis) (4, 32, 43). Emboli to the MCA were found to result in iSCI if the peripheral tissue is preserved by an outstanding collateral system, and in combination with/or if the occlusion is of transient nature, which might be due to spontaneous thrombolysis, IV rtPA induced thrombolysis (23) or, as shown here, with ET. In contrast to the reported recanalization rate of 78.2% within the presented study, early recanalization following IV rtPA was found to occur in approximately one-third of proximal MCA occlusions (44). The incidence of iSCI may thus increase considerably with the spread of endovascular therapy for LVOs.

It is plausible that iSCI patients had less neurological sequelae because all parts of the eloquent neocortex were spared. The generally more favorable outcome of patients with iSCI corroborates results from a meta-analysis of patients with iSCI reviewed by Caplan (45). On the other hand, it is evident that most patients with iSCI suffer from, albeit smaller, neurological symptoms, and some patients are functionally disabled. Further analyses of patients with this ischemic stroke pattern may elucidate underlying pathophysiology or imaging correlates explaining this heterogeneity [e.g., grade of capsule ischemia and preexisting white matter lesions (20)]. Furthermore, patients with iSCI were found to frequently suffer from different degrees of less conspicuous neuropsychological dysfunctions (4, 46). This may be of relevance as more than half of the acute stroke patients who achieve an excellent functional outcome suffer from cognitive impairment, depression, or participation restriction within 3 years after the acute event (47).

The current study suggests that the once rare iSCIs are likely to increase in incidence as the number of endovascular thrombectomies increases. Therefore, these patients warrant further study. It is likely, given the isolated involvement of subcortical structures, that these patients will require tailored subacute therapy and rehabilitation strategies. This may have significant implications for far ranging ischemic stroke services from the acute stroke unit to community-based rehabilitation and workplace reintegration. Furthermore, the increased incidence may allow for a more detailed pathophysiological analysis of ischemia confined to parts of the putamen, the caudate nucleus, or the internal capsule (e.g., subcortical aphasia, apathy, etc.).

The present study has a number of limitations, in general due to its retrospective nature: A considerable number of patients were excluded due to missing or inadequate imaging follow-up. This was especially an issue for patients treated before mid-2012, when institutional protocols required less imaging in patients with good neurological outcome. This may be a source of attrition bias. Second, CT-derived definition of iSCI is susceptible to underestimation of infarct extent, which may introduce a detection bias for peripheral ischemia. However, CT-derived definitions of iSCI were based upon CTs on days 3–4 and were evaluated on 2.5 mm slices, reducing the rates of false positive ratings. Furthermore, outcome effects were observed independent of the imaging modality, on which the iSCI definition relies on (see Figure S1 in Supplementary Material). As both patients with iSCI and niSCI had a better outcome if they received an MRI scan instead of a CT scan (probably because MRI is clinically more feasible in patients doing well), it is unlikely that all heterogeneity within the iSCI group is introduced by false positive rating of iSCI in patients receiving a CT scan. Finally, functional mid-term outcome data were available only for 150/206 patients.

## Conclusion

Successful reperfusion in patients presenting with a proximal MCA occlusion and robust collaterals markedly favors occurrence of iSCIs. Considering the widespread use of ET in clinical practice, the incidence of this stroke pattern is likely to rapidly rise. The patient group with iSCI was found to have a very benign, albeit heterogeneous, short- and mid-term clinical course. Further research may elucidate the pathophysiological role and corresponding imaging correlates of isolated striatocapsular ischemia and may further initiate a discussion about deliberate follow-up and rehabilitation strategies for this particular patient group.

## Ethics Statement

Written consent was waived by the local ethics committee due to the retrospective design of the present study. Analyses were approved by the “Ethikkomission der Fakultat fuer Medizin der Technischen Universitat Muenchen” (http://www.ek-med-muenchen.de).

## Author Contributions

JK: concept, statistical analysis, image interpretation, data acquisition, interpretation, and initial manuscript draft. TH, KB, and SW: data acquisition, interpretation, and critical revision of the manuscript. ML: data acquisition and critical revision of the manuscript. CZ: critical revision of the manuscript and study supervision. TB-B: data interpretation and critical revision of the manuscript. NM: writing of the initial manuscript draft, data interpretation, and statistical analysis. JK: study supervision, statistical analysis, data acquisition, interpretation, and critical revision of the manuscript.

## Conflict of Interest Statement

The authors declare that the research was conducted in the absence of any commercial or financial relationships that could be construed as a potential conflict of interest.
