# Supplementary figures and images for: Isolated Striatocapsular Infarcts after Endovascular Treatment of Acute Proximal Middle Cerebral Artery Occlusions: Prevalence, Enabling Factors, and Clinical Outcome
Source: Front Neurol. 2017 Jun 19;8:272. doi: 10.3389/fneur.2017.00272 (PMC5474958; doi:10.3389/fneur.2017.00272)

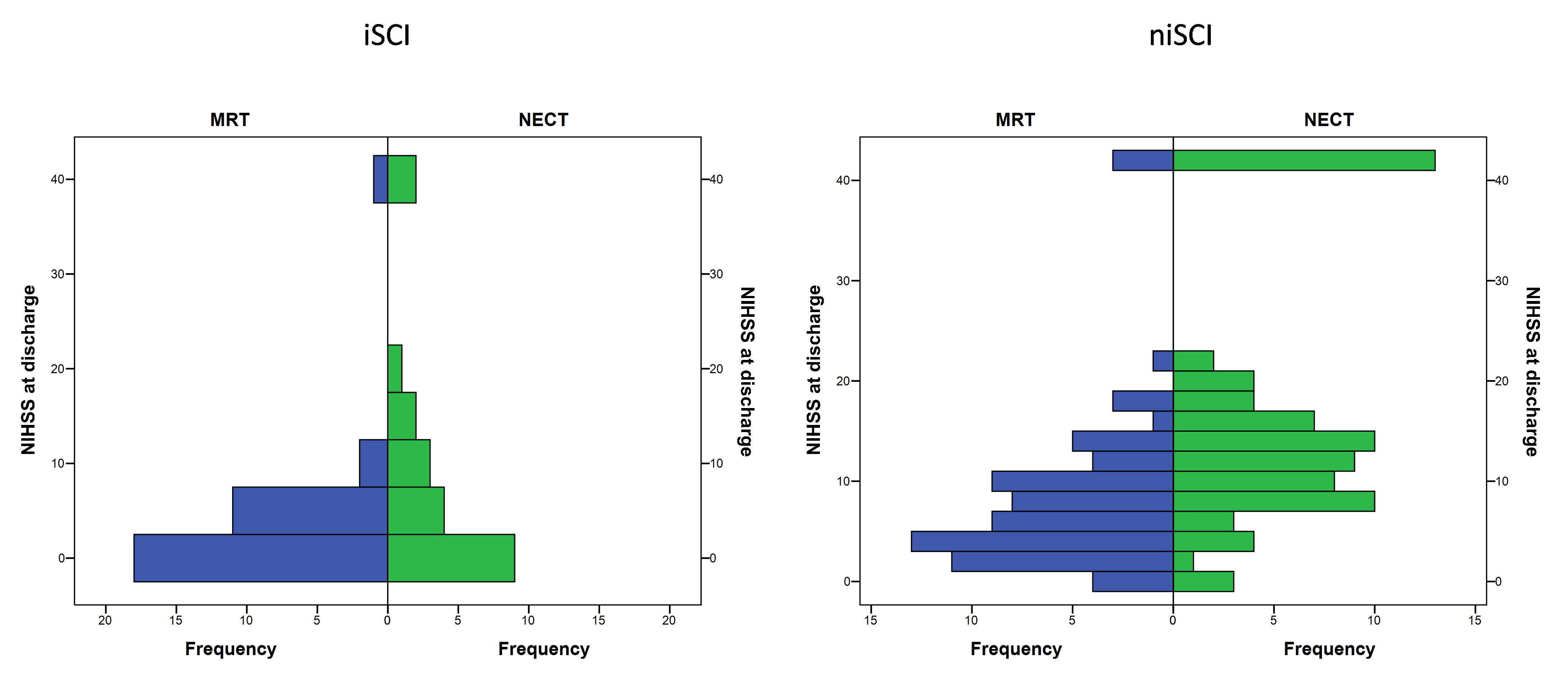

Supplement: Supplementary file 1 [file image_1.tif]
